# Efficient Open Fermentative Production of Polymer-Grade L-Lactate from Sugarcane Bagasse Hydrolysate by Thermotolerant *Bacillus* sp. Strain P38

**DOI:** 10.1371/journal.pone.0107143

**Published:** 2014-09-05

**Authors:** Lili Peng, Nengzhong Xie, Ling Guo, Limin Wang, Bo Yu, Yanhe Ma

**Affiliations:** 1 CAS Key Laboratory of Microbial Physiological and Metabolic Engineering, Institute of Microbiology, Chinese Academy of Sciences, Beijing, China; 2 National Engineering Research Center for Non-food Biorefinery, Guangxi Academy of Science, Nanning, China; 3 Tianjin Institute of Industrial Biotechnology, Chinese Academy of Sciences, Tianjin, China; University of Nottingham, United Kingdom

## Abstract

Lactic acid is one of the top 30 potential building-block chemicals from biomass, of which the most extensive use is in the polymerization of lactic acid to poly-lactic-acid (PLA). To reduce the cost of PLA, the search for cheap raw materials and low-cost process for lactic acid production is highly desired. In this study, the final titer of produced L-lactic acid reached a concentration of 185 g·L^−1^ with a volumetric productivity of 1.93 g·L^−1^·h^−1^ by using sugarcane bagasse hydrolysate as the sole carbon source simultaneously with cottonseed meal as cheap nitrogen sources under the open fed-batch fermentation process. Furthermore, a lactic acid yield of 0.99 g per g of total reducing sugars was obtained, which is very close to the theoretical value (1.0 g g^−1^). No D-isomer of lactic acid was detected in the broth, and thereafter resulted in an optical purity of 100%, which exceeds the requirement of lactate polymerization process. To our knowledge, this is the best performance of fermentation on polymer-grade L-lactic acid production totally using lignocellulosic sources. The high levels of optically pure l-lactic acid produced, combined with the ease of handling and low costs associated with the open fermentation strategy, indicated the thermotolerant *Bacillus* sp. P38 could be an excellent candidate strain with great industrial potential for polymer-grade L-lactic acid production from various cellulosic biomasses.

## Introduction

Lactic acid is an important chemical that exhibits a wide range of potential applications in food and non-food industries, including cosmetic, pharmaceutical, and chemical industries. The demand for lactic acid has been increasing considerably owing to the promising applications of its polymers, poly(L-lactic-acid) (PLA), as an environmentally friendly alternative to petrochemical plastics. The physical properties and stability of PLA depend on the isomeric composition of the lactic acid used in its synthesis; consequently, it is essential to ensure optical purity of the L-lactic acid reagent prior to polymerization [1]. Currently, the commercial production of L-lactic acid is based on microbial fermentation of starch sugar. To reduce the cost and increase the economy of lactic acid production, development of an efficient and cost-effective process for lactic acid fermentation that utilizes inexpensive, non-food substrates is highly desired [2].

Lignocellulosic biomass is a potential feedstock because they are cheap, abundant and renewable, and do not compete with food. The efficient bioconversion of lignocelluloses-derived sugars to lactic acid is a key challenge for economically feasible fermentation processes. Among the abundant lignocellulosic biomass, sugarcane bagasse, an abundant byproduct of the sugar production industry in the southern China, primarily consists of 43.6% cellulose, 33.8% hemicellulose, and 18.1% lignin [3]. Sugarcane bagasse hydrolysate mainly consists of fermentable sugars, such as glucose and xylose, and is a renewable, readily available raw material that could be used for large-scale production of lactic acid. Although some studies have investigated the potential of utilizing lignocellulosic biomass as carbon sources, such as sugarcane bagasse, and yeast extract (YE) as nitrogen source, the lactic acid concentration produced using the present processing technologies is only around 40–70 g·L^−1^ [4], [5], which is far below the requirement for industrial organic acid production (generally above 100 g·L^−1^) [6]. Furthermore, use of expensive nitrogen sources is a major limitation for developing a cost-effective lactic acid production method. Among the various complex nitrogen sources, YE is the best choice for both microbial growth and lactic acid production [7]. The nitrogen source, such as YE, account for 38% of the total fermentation cost during lactic acid fermentation [8], and is a major factor affecting the economy of lactic acid production. To develop an economically viable industrial process, both productivity and cost must be considered at the same time, which also implies a need for renewable and cheaper alternative raw materials to substitute for YE. Until now, various low-cost nitrogen materials such as soy protein hydrolysates [9], defatted rice bran [10], and Baker's yeast cells [8], have been investigated as YE substitutes during lactic acid production. However, most of these materials were relatively ineffective. As a high final titer is important for reduction of the overall separation and concentrating costs of lactic acid, identifying robust lactic acid producers and efficient fermentation process that provide higher titers is necessary. Cotton is abundant in China and the cottonseed left behind after processing is considered an agricultural waste, thus the cost of cottonseed is far less than commercial YE. Additionally, cottonseeds are rich in proteins, amino acids and vitamins that are conducive for the growth of lactic acid bacteria. Thus, the material offers promise as a low-cost feedstock for chemicals production.

In addition to inexpensive substrates, low-cost operation processes are also essential for reducing the production costs. The use of nonsterile conditions for industrial fermentation reduces equipment needs, energy consumption, and labor costs [11]. The use of nonsterile conditions also inhibits the Maillard reaction, which leads to the production of unfavorable furfural compounds and subsequently increases the coloration of the fermentation broth [12]. Furthermore, development of an economically viable l-lactic acid production process could therefore compete with the traditional production methods [13].

The aim of this study is to develop a cost-effective process for L-lactic acid production that uses inexpensive raw materials and a low-cost fermentation process. High yields and titer of L-lactic acid were obtained by using sugarcane bagasse hydrolysate and cottonseed as inexpensive carbon and nitrogen sources, respectively, and by using *Bacillus* sp. P38 for conducting open fermentation, which should significantly reduce the cost of the lactate production.

## Materials and Methods

### Strain and chemicals

*Bacillus* sp. P38 was inoculated in medium containing (per liter) 50 g of glucose, 10 g of yeast extract, and 30 g of calcium carbonate. The seed culture was prepared as follows: a loop of cells from the fully grown slant was inoculated into 50 mL of the above mentioned sterile medium in 100-mL conical flasks and incubated at 50°C for 24 h without agitation. Cottonseed, a dried powder that contains 8% (w/w) total nitrogen, was purchased from Qingdao Create Medium Co., Ltd (Qingdao, China). Commercially available neutral protease (EC 3.4.24.28), with an enzyme activity of 5×10^4^ U·g^−1^, was purchased from Novozymes (Denmark). All other chemicals were of analytical grade and were commercially available.

### Procedures for preparing sugarcane bagasse hydrolysate

The sugarcane bagasse hydrolysate was prepared by using an acid-base-enzyme joint treatment process, which was referenced from previous reports after minor modifications [14], [15]. The hydrolysis procedure used was as follows. 1) Pretreatment: the sugarcane bagasse was powdered, and the particles sized ≤1 mm were collected after sieving the powder. 2) Dilute sulfuric acid treatment: 500 g powder was thoroughly mixed with 5 L of 1.5% sulfuric acid and autoclaved at 121°C for 1 h to release the sugars from hemicellulose. The hydrolysate recovered after acid hydrolysis was separated into a solid and a liquid fraction by filtration. 3) Dilute alkaline hydrolysis: the solid fraction was mixed with 5 L of 1.5% sodium hydroxide, and autoclaved at 100°C for 1 h to remove lignin in sugarcane bagasse. The filter cake was washed with water. 4) Cellulose hydrolysis: The filter cake was mixed with 2 L of 20 mM citrate buffer (pH 4.8), and enzymatic hydrolysis experiments were carried out by using 5×10^6^ U commercial cellulase (Imperial Jade Bio-Technology Company, China) at 50°C for 48 h. The hydrolysate was then concentrated by rotary evaporator to give the final concentration of 355 g·L^−1^ glucose and 37 g·L^−1^ xylose with 0.4 g·L^−1^ acetic acid and 0.56 g·L^−1^ lignin. No 2-furfural and formic acid were detected in the concentrated hydrolysate.

### The effects of lignin on fermentation capacity of strain P38

To investigate the lignin tolerance, strain P38 was cultivated with different initial concentrations of lignin in the medium with (per liter) 20 g glucose, 10 g yeast extract and 12 g calcium carbonate. The initial glucoses concentration was set at 20 g·L^−1^ to reduce the fermentation time to give more reliable results for lignin tolerance. The lignin concentration varied from 0 to 10 g·L^−1^. The glucose consumption and l-lactic acid production were analyzed. All experiments were conducted in 100 mL flasks containing 50 mL medium at 50°C for 24 h without agitation.

### Optimization of fermentation conditions

Preliminary studies were conducted to determine the optimal conditions required for promoting lactic acid production. A simultaneous hydrolysis and fermentation method was used. To determine the optimum protease concentration for fermentation, a medium with the following composition was used: cottonseed meal, 40 g·L^−1^; glucose, 72 g·L^−1^; and CaCO_3_, 48 g·L^−1^. Various concentrations of neutral protease (0, 0.1, 0.2, 0.3, 0.4, 0.5 or 1.0 g·L^−1^) were evaluated to determine the optimum concentration. The neutral protease solution was added to medium after filtration. To determine the optimal cottonseed meal concentration, a medium of the following composition was used: glucose, 72 g·L^−1^; neutral protease, 0.3 g·L^−1^; and CaCO_3_, 48 g·L^−1^. The following initial cottonseed meal concentrations were used: 10, 20, 30, 40, 50, or 60 g·L^−1^. To determine the optimal initial total reducing sugar concentration, a range of initial total reducing sugar concentrations of 54.5, 66.5, 90.1, 119.0, and 180.9 g·L^−1^ were used. The toxicity of lactic acid for bacterial cell growth is the key obstacle for the lactic acid fermentation. CaCO_3_ is normally added during fermentation to neutralize lactic acid to reduce the inhibitory effects of high concentration lactic acid on cell growth and productivity. In this study, CaCO_3_ 60% (w/w) of the total reducing sugars, was added to the medium to maintain the pH at about 5.5 [16].

### Fed-batch fermentation under open fermentation conditions

Fed-batch fermentations were performed in a 1-L fermentor with an initial working volume of 500 mL optimized medium at 50°C with an agitation speed of 50 rpm without aeration and under unsterile conditions. The experiment was performed with an inoculum volume of 30% (v/v). The initial concentration of the total reducing sugars was set at approximately 85 g·L^−1^. When the concentration of residual sugars decreased to approximately 20 g·L^−1^, sugars with 60% CaCO_3_ (w/w) were fed to bring the sugar concentration to approximately 50 g·L^−1^ four times. The broth pH could be maintained at about 5.5 by adding 60% CaCO_3_ of the added sugars (w/w). The yield was defined as produced lactic acid per consumed sugars from hydrolysate (w/w), which is consistent with previous reports [16], [17].

### Analytical methods

The glucose and L-lactate concentrations were measured with the SBA-40D biosensor analyzer, based on the specific enzymatic reaction (Institute of Biology, Shandong Academy of Sciences, China) [16]. The total concentration of the reducing sugars was measured by using a SGD-IV automatic analyzer, based on the traditional DNS methods (Institute of Biology, Shandong Academy of Sciences, China) [17]. The optical purity of L-lactic acid was determined at 254 nm by using high-performance liquid chromatography (HPLC) system equipped with a chiral column (4.6 mm×50 mm, MCI GEL CRS10 W, Japan). The mobile phase was 2 mM CuSO_4_ at a flow rate of 0.5 mL·min^−1^ (25°C). The optical purity of L-lactic acid was defined as follows: L-lactic acid/(L-lactic acid + D-lactic acid)×100%.

## Results and Discussion

### Fermentation inhibitor tolerance of strain P38

**Figure 1:**
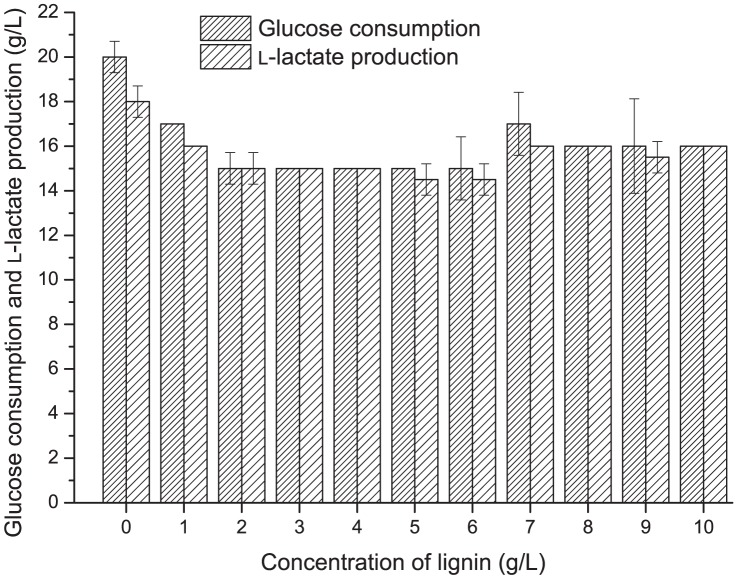
The fermentation inhibitor tolerance capacities of *Bacillus* sp. P38 under different concentrations of lignin. The experiments were carried out in 100 mL flasks containing 50 mL of the fresh medium with (per liter) 20 g glucose, 10 g yeast extract and 12 g calcium carbonate. The error bars in the figure indicate the standard deviations of three parallel replicates.

During the sugarcane bagasse hydrolysis process, some soluble materials such as lignin, acetic acid, and 2-furfural are also produced, which can inhibit both cell growth and sugar utilization during the fermentation process [15]. *Bacillus* sp. P38 was shown to be an excellent strain that tolerated high concentrations of fermentation inhibitors, such as 2-furfural (up to 10 g·L^−1^), vanillin, and acetic acid (>6 g·L^−1^) in the hydrolysate [16]. As lignin in sugarcane bagasse hydrolysate has been previously reported to be a fermentation inhibitor [18], experiments were first conducted to test the lignin tolerance capacity of *Bacillus* sp. P38. We showed that the high lignin concentrations had no significant effects on glucose consumption and lactate production. Only a 5% decrease of glucose consumption and lactate production occurred at the high lignin concentration of 10 g·L^−1^ (Fig. 1), which is much higher than the normal lignin concentrations in hydrolysate (<1 g·L^−1^). Thus, *Bacillus* sp. P38 can be used for efficient lactate production from sugarcane bagasse hydrolysate.

### Optimal fermentation conditions for L-lactic acid production

**Figure 2:**
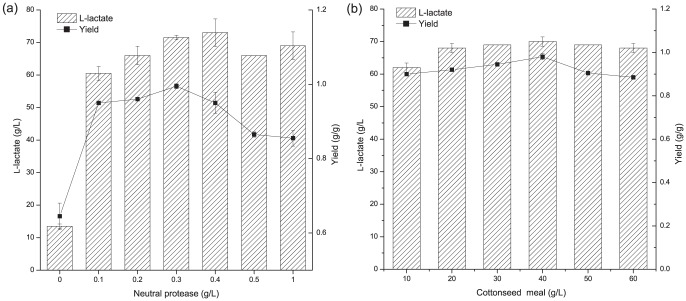
The effects of neutral protease and cottonseed concentrations for L-lactic acid production. (a) The effect of the various neutral protease concentrations. (b) The effect of cottonseed concentrations. The error bars in the figure indicate the standard deviations of three parallel replicates.

The nitrogen source can have a strong positive effect on cell growth, as it can accelerate the growth of strains and allow them to reach higher cell densities [19]. The preliminary test was first conducted to investigate the role of cottonseed in the fermentation. The main nutritive ingredients in cottonseed powder used in this study are 51.32% protein, 1.89% fat and 5.34% cellulose. As no cellulase or other chemical treatments were applied in the fermentation process, the released total reducing sugars from cottonseed powder (40 g L^−1^) was determined to be just 0.39 g L^−1^ initially. To further investigate the possibility if strain P38 could cleavage cellulose directly during the fermentation, 40 g L^−1^ cottonseed with 10 g L^−1^ NH_4_Cl and 0.3 g L^−1^ neutral protease were used as fermentation medium to test the capacity of lactic acid production. The final titer of produced lactic acid in 96 h was<0.1 g L^−1^ (after subtracting the initial concentration of lactic acid from seed culture) and the cell growth was also very weak. The above results clearly indicated that cottonseed served as nitrogen source but not as carbon source in the fermentation. Then the simultaneous hydrolysis and fermentation strategy was applied to L-lactic acid fermentation in this study. The neutral protease was added initially to gradually release the available nitrogen source for cell growth and lactate production. As shown in Fig. 2a, L-lactic acid concentration initially increased in proportion with the amount of neutral protease added. In the absence of neutral protease, the lactic acid production was substantially low (13.5 g·L^−1^). The highest lactate yield and equivalent titer of 70 g·L^−1^ was obtained at neutral protease concentration of 0.3 g·L^−1^.

**Figure 3:**
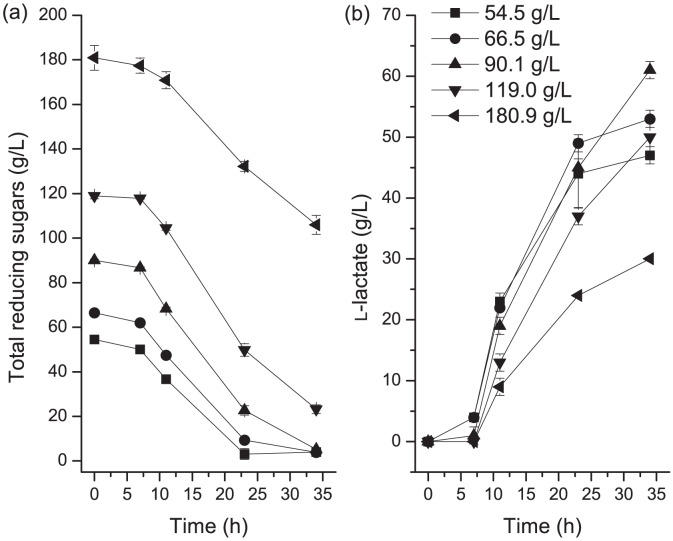
The effects of initial total reducing sugars for L-lactic acid production. (a) Sugar consumption and (b) L-Lactate production. The error bars in the figure indicate the standard deviations of three parallel replicates.

Next, we determined the optimum concentration of cottonseed required for efficient lactate production. Maximum L-lactic acid production (70 g·L^−1^) was obtained with a yield of 0.98 g·g^−1^ glucose at 40 g·L^−1^ cottonseed. Since L-lactate yields were lower at cottonseed concentrations that were higher than 40 g·L^−1^, cottonseed concentration of 40 g·L^−1^ was determined to be the optimum concentration (Fig. 2b). The initial total reducing sugar concentration was also optimized and the finally chosen value for subsequent fed-batch fermentation was 90 g·L^−1^, because it produced the highest lactate yield and induced the highest sugar consumption rate (Fig. 3).

Cellulose- and hemicellulose-derived carbohydrate feedstocks contained a variety of mixed sugars, mainly glucose and xylose. In order to maximize lactic acid yield and production, complete utilization of mixed sugars is essential. Carbon catabolite repression (CCR) is a common phenomenon in bacteria and very few bacteria have been reported which consume different sugars simultaneously [20]. Therefore, for industrialization of lactic acid production from cellulosic materials, it is desirable to use CCR-negative strain for lactic acid production from mixed sugar substrates. *Bacillus* sp. strain P38 could utilize both the two sugars simultaneously [16], proving its feasibility for L-lactic acid production from low-cost raw materials.

### L-Lactic acid production from bagasse hydrolysate by fed-batch fermentation

**Figure 4:**
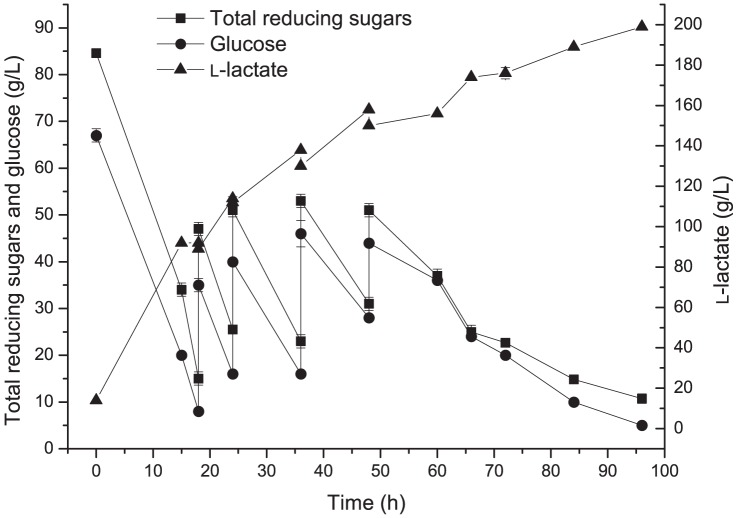
Open fed-batch fermentation from bagasse hydrolysate in fermentor. Symbols represent carbohydrates consumption and L-lactic acid production in the fermentation medium (g L^−1^): Total reducing sugars (▪), Glucose (•), and L-lactic acid (▴). The error bars in the figure indicate the standard deviations of three parallel replicates.

The fed-batch fermentation was conducted under open fermentation conditions to reduce the processing costs. The 30% (v/v) inoculum was applied to lower the risk of bacterial contamination under the unsterile fermentation conditions and also increase the initial productivity. The lactic acid concentration in broth at the end of the open fermentation processing was determined to be 199 g·L^−1^. After subtracting the initial lactic acid concentration (14 g·L^−1^) brought from the seed culture, 185 g·L^−1^ lactic acid was produced in the fermentation process with a high yield of 99% and a high average productivity of 1.93 g·L^−1^·h^−1^ (Fig. 4). As no cellulose or other chemical treatments were applied in the process, the released total reducing sugars from cottonseed powder was rather low (0.39 g·L^−1^), which could not have obvious effects on the calculated production yield. No D-isomer of lactic acid was detected in the broth, resulted in a 100% optical purity, which exceeds the requirement of lactate polymerization process (optical purity >99%).

Large quantities of lignocellulosic wastes are produced globally every year. Bioconversion of lignocellulosic biomass could significantly reduce the production of organic chemical waste products. Thus, abundantly available agricultural by-products, such as sugarcane bagasse, could be used as an inexpensive substrate in the biotechnological production process [21]. Previously evaluated *Lactobacillus* strains have yielded low lactate titers. Laopaiboon et al. [15] used detoxified sugarcane hydrolysate supplemented with 7 g·L^−1^ xylose and 7 g·L^−1^ yeast extract for lactic acid fermentation, which produced only 10.85 g·L^−1^ lactic acid with equivalent concentrations of acetic acid, formic acid, and ethanol. Under simultaneous saccharification and fermentation process, a maximum lactic acid concentration of 67 g·L^−1^ was produced from bagasse cellulose (80 g·L^−1^) by a *Lactobacillus delbrueckii* mutant (Uc-3), with a productivity of 0.93 g·L^−1^·h^−1^ and a yield of 0.83 g·g^−1^ [5]. A previous study showed that 2.9 g of L-lactic acid per 5 g of initial substrate was obtained. As the sugarcane bagasse was soaked with cassava starch hydrolysate containing 3 g reducing sugar in a solid-state condition, which resulted to 97% conversion rate of sugar to lactic acid [22].

As *Lactobacillus* strains have been shown to inefficiently utilize lignocellulose hydrolysates, other bacterial strains are being evaluated for lactic acid production efficacy. *Thermoanaerobacterium aotearoense* was engineered to produce L-lactic acid from xylose, while the highest titer was around 45 g L^−1^ with a yield of 89% from mixed sugars of glucose and xylose [2]. Thermotolerant *Bacillus* species, which could grow at above 50°C conditions so as to conduct the fermentation under unsterile conditions, attracted more and more attention for lactic acid production recently while the production titers were not satisfactory for most of cases. A *Bacillus* strain has been shown to produce 55 g L^−1^ L-lactic acid from sugarcane bagasse hemicellulose hydrolysate with a yield of 89% [4]. *Bacillus* sp. strain 36D1 produced 35 g·L^−1^ lactic acid in 144 h under a process of simultaneous saccharification and co-fermentation of crystalline cellulose and sugarcane bagasse hydrolysate [23]. As a high final titer is important for reduction of the overall costs of lactic acid, strain and process improvement to get higher fermentation performance is still an urgent target [24]. *Bacillus* sp. P38 was previously shown to be an efficient lactic acid producer from corn stover hydrolysate and yeast extract under sterile fermentation conditions [16]. Additionally, *Bacillus* sp. P38 is a thermotolerant lactate producer, the medium sterilization is not necessary to avoid contamination during fermentation under 50°C. This study showed that *Bacillus* sp. P38 could also efficiently produce lactic acid from other inexpensive substrates such as sugarcane bagasse hydrolysate with cottonseed as cheap nitrogen source, and that open fermentation produced a high lactic acid concentration (185 g·L^−1^) with a high yield of 0.99 g·g^−1^ and 100% optical purity, which is beyond the requirement for industrial organic acid production (above 100 g·L^−1^). It should be notable that the price of cottonseed powder is only 1/10 of that of yeast extract and equivalent performance was obtained in this study. In addition, the open fermentation strategy applied in this study further lowered the production costs, which made its industrialization very promising. The efficient L-lactic acid production from totally inexpensive and abundantly available agricultural wastes by using *Bacillus* sp. P38 indicates its significant industrial potential.

## Conclusions

The process for obtaining high L-lactic acid concentration and yield was developed from sugarcane bagasse hydrolysate and cottonseed powder under totally unsterile fermentation conditions. Due to the thermotolerant feature of *Bacillus* sp. P38, the risk of contamination by other strains could be avoided under such fermentation temperature (50°C) and high inoculum volume (30%, v/v). The high tolerance of *Bacillus* sp. strain P38 to the toxicity of fermentation inhibitors indicates that this bacterial strain could be useful for developing an efficient and economical fermentation process for producing L-lactic acid from various cellulosic biomasses.
